# Nanoscale click-reactive scaffolds from peptide self-assembly

**DOI:** 10.1186/s12951-017-0300-7

**Published:** 2017-10-06

**Authors:** Alexander P. M. Guttenplan, Laurence J. Young, Dijana Matak-Vinkovic, Clemens F. Kaminski, Tuomas P. J. Knowles, Laura S. Itzhaki

**Affiliations:** 1 Department of Pharmacology, University of Cambridge, Tennis Court Road, Cambridge, CB2 1PD UK; 2 Department of Chemistry, University of Cambridge, Lensfield Road, Cambridge, CB2 1EW UK; 3 Department of Chemical Engineering and Biotechnology, University of Cambridge, Philippa Fawcett Drive, Cambridge, CB3 0AS UK

**Keywords:** Amyloid fibrils, Click reactions, Bioconjugation, Super-resolution microscopy, Self-assembly, Bionanotechnology

## Abstract

**Background:**

Due to their natural tendency to self-assemble, proteins and peptides are important components for organic nanotechnology. One particular class of peptides of recent interest is those that form amyloid fibrils, as this self-assembly results in extremely strong, stable quasi-one-dimensional structures which can be used to organise a wide range of cargo species including proteins and oligonucleotides. However, assembly of peptides already conjugated to proteins is limited to cargo species that do not interfere sterically with the assembly process or misfold under the harsh conditions often used for assembly. Therefore, a general method is needed to conjugate proteins and other molecules to amyloid fibrils after the fibrils have self-assembled.

**Results:**

Here we have designed an amyloidogenic peptide based on the TTR105-115 fragment of transthyretin to form fibrils that display an alkyne functionality, important for bioorthogonal chemical reactions, on their surface. The fibrils were formed and reacted both with an azide-containing amino acid and with an azide-functionalised dye by the Huisgen cycloaddition, one of the class of “click” reactions. Mass spectrometry and total internal reflection fluorescence optical microscopy were used to show that peptides incorporated into the fibrils reacted with the azide while maintaining the structure of the fibril. These click-functionalised amyloid fibrils have a variety of potential uses in materials and as scaffolds for bionanotechnology.

**Discussion:**

Although previous studies have produced peptides that can both form amyloid fibrils and undergo “click”-type reactions, this is the first example of amyloid fibrils that can undergo such a reaction after they have been formed. Our approach has the advantage that self-assembly takes place before click functionalization rather than pre-functionalised building blocks self-assembling. Therefore, the molecules used to functionalise the fibril do not themselves have to be exposed to harsh, amyloid-forming conditions. This means that a wider range of proteins can be used as ligands in this process. For instance, the fibrils can be functionalised with a green fluorescent protein that retains its fluorescence after it is attached to the fibrils, whereas this protein loses its fluorescence if it is exposed to the conditions used for aggregation.

**Electronic supplementary material:**

The online version of this article (doi:10.1186/s12951-017-0300-7) contains supplementary material, which is available to authorized users.

## Background

Peptide self-assembly has emerged as an attractive route towards organic nanotechnology. Such self-assembled designed biomaterials have found a range of uses from templating nano- and microscale electronics to acting as scaffolds for cell culture or tissue engineering [1–3]. A particularly simple yet general form of peptide and protein self-assembly is that leading to linear, homomolecular structures; in many cases, the component peptides within these nanostructures are held together with a backbone–backbone hydrogen bonding network that leads to extended β-sheets running along the fibril axis [4, 5]. Such β-sheet-rich amyloid or amyloid-like fibrils are recognised for their biological roles both in diseases such as Alzheimer’s and Parkinson’s [6, 7] and as functional components in some organisms [8–11].

More recently, amyloid fibrils have been explored as potential materials for use in nanotechnology applications. One reason for this interest is their robust mechanical strength and rigidity, which have some of the highest values known for biological systems [12–14]. In addition, the fact that fibrils self-assemble has led to research into making them functional, for example as electrically conducting nanowires [15, 16], for carbon capture [17] or as drug carriers [18]. However, introducing chemical functionality to these self-assembled biomaterials has hitherto often required the material to self-assemble from components that already include the functional moiety. In this approach, the choice of functionality is limited to species that can survive the conditions of self-assembly with their function intact and that do not themselves disturb it [19]. Here, we address this limitation with a new method, which allows the fibrils to be functionalized after they are formed using mild conditions that tolerate a wide range of substrates. We show that this approach allows even sensitive proteins which are not compatible with the self-assembly conditions to be used as the cargo and that this strategy thus significantly extends the chemical space of available for selecting species that can be organized on the nanoscale through peptide self-assembly. Moreover, the strategy is highly adaptable with the potential for proteins to be attached to fibrils via a non-natural amino acid inserted at any position in the protein (using a variety of methods [20, 21]) or for the same protein molecule to be attached to multiple fibrils, in contrast with existing methods where the protein can only be attached at one terminus [22]. Our approach will allow the amyloid fibrils to be linked together by proteins so that a force exerted on the amyloid fibrils, for instance by deformation of an amyloid-based material [13, 23], is transmitted to the protein as both termini of the protein are connected to the fibril rather than one being free. This would allow force-responsive proteins to be incorporated in an amyloid-based material for soft nanotechnology applications. Also, it means that proteins can be conjugated to fibrils at a different position in cases where terminal conjugation would inhibit the function of the protein.

To demonstrate the general nature of our approach, we generated a nanoscale scaffold capable of capturing a wide range of functional molecules, including folded proteins and small molecules, to pre-assembled fibrils. We exploited the Huisgen cycloaddition [24], one of the class of “click” chemical reactions defined by Sharpless et al. [25] which have a wide range of applications in bioconjugation [26]. In addition to the defining features of a “click” reaction—proceeding to completion in water at relatively low temperatures without side products—the Huisgen reaction is completely bioorthogonal, as it takes place between functional groups that do not exist in natural biomolecules [27]. Therefore, it can be used to selectively link together biomolecules into which the reactive functional groups have been introduced.

**Fig. 1:**
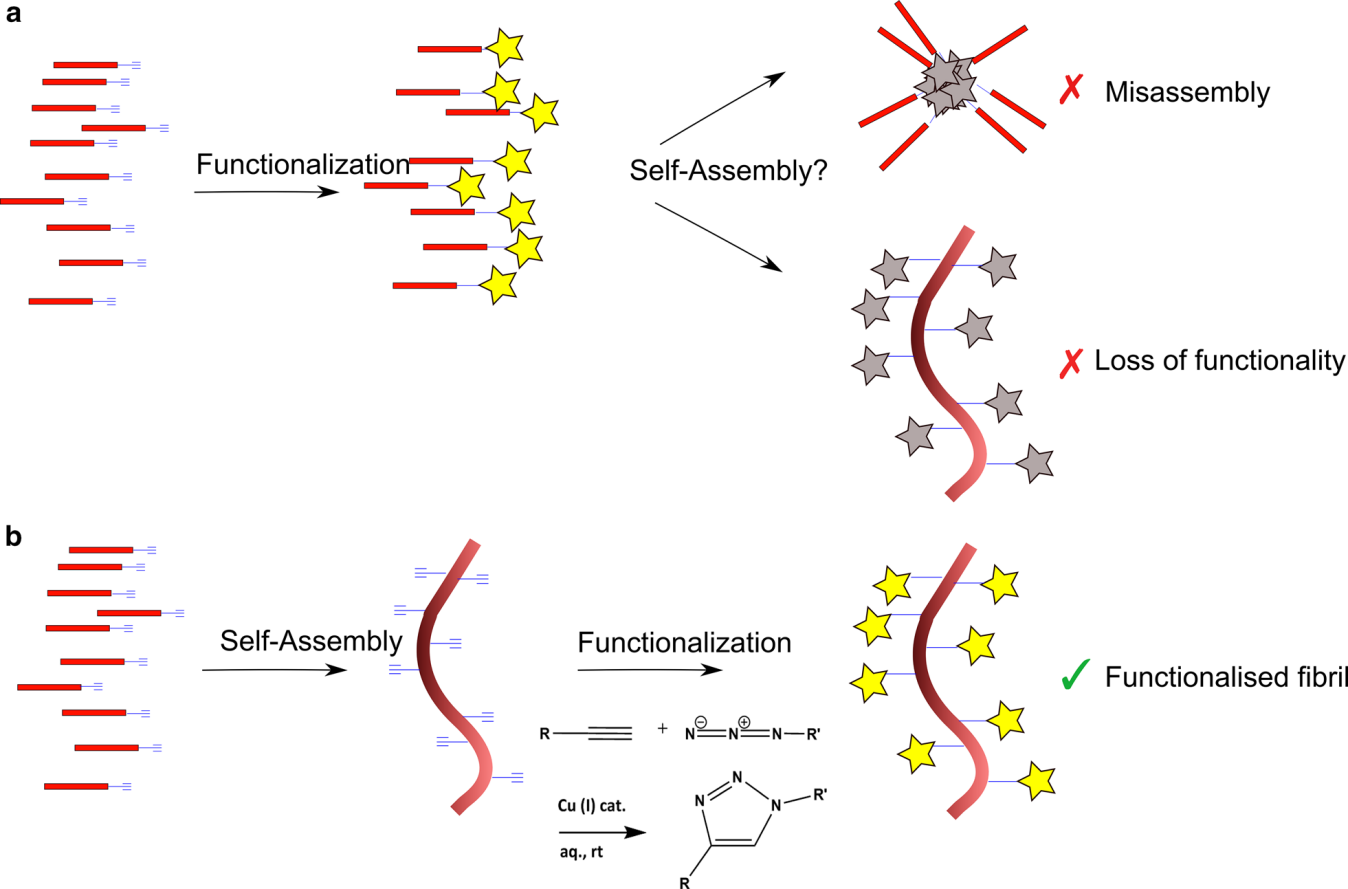
Pre-assembly functionalization versus post-assembly functionalization. **a** In the conventional route, molecule building blocks are functionalized prior to self-assembly. In such cases, functional moieties can, however, prevent fibril self-assembly or lose their functionality. **b** The method detailed in this paper avoids exposing the functional species to amyloid-forming conditions and results in the formation of a click-reactive scaffold that can be functionalized after nanoscale self-assembly has taken place

This reaction has previously been used to produce amyloid or other beta-sheet-rich fibrils functionalized with oligonucleotides [28] or polymers [29]. However, in both of these cases the cycloaddition reaction was performed on the peptide monomers, which were then allowed to aggregate to form the fibril. To the best of our knowledge the present work represents the first example of a “click” reaction being performed on an amyloid fibril after it has been formed (Fig. 1). Crucially, functionalizing the nanofibrils after they have been formed rather than before means that the species used to functionalize them need not be exposed to aggregation-promoting conditions. This approach thus has the important advantage of allowing a greater range of species, particularly proteins, to be used to functionalize amyloid fibrils, as proteins can be used that would otherwise denature and form aggregates under the conditions in which the fibrils form.

The amyloid-forming peptide used here was based on the well-characterized TTR105-115 peptide [30], which is a fragment of the human plasma protein transthyretin—itself a protein that forms amyloid associated with diseases such as familial amyloid polyneuropathy [31]. Fibrils formed from the TTR105-115 fragment are, however, non-toxic and biocompatible and have been explored for tissue culture applications [32]. The TTR105-115 fragment is known to form amyloid fibrils which can display chemical functionality at the N-terminal end of the peptide on their surface [32]. The peptide was chosen because it is short enough to synthesize easily by solid-phase peptide synthesis. This method allows the incorporation of almost any chemical functionality via non-natural amino acids, as the amino acids used need not be viable substrates for ribosomal protein synthesis. However, it is long enough that the amyloidogenic part of the peptide is significantly larger than the non-amyloidogenic part used to add click functionality. The latter consists of a 3-glycine linker followed by *O*-propargyl serine—an alkyne-containing amino acid chosen for ease of synthesis.

## Results

### Synthesis of fibrils

**Fig. 2:**
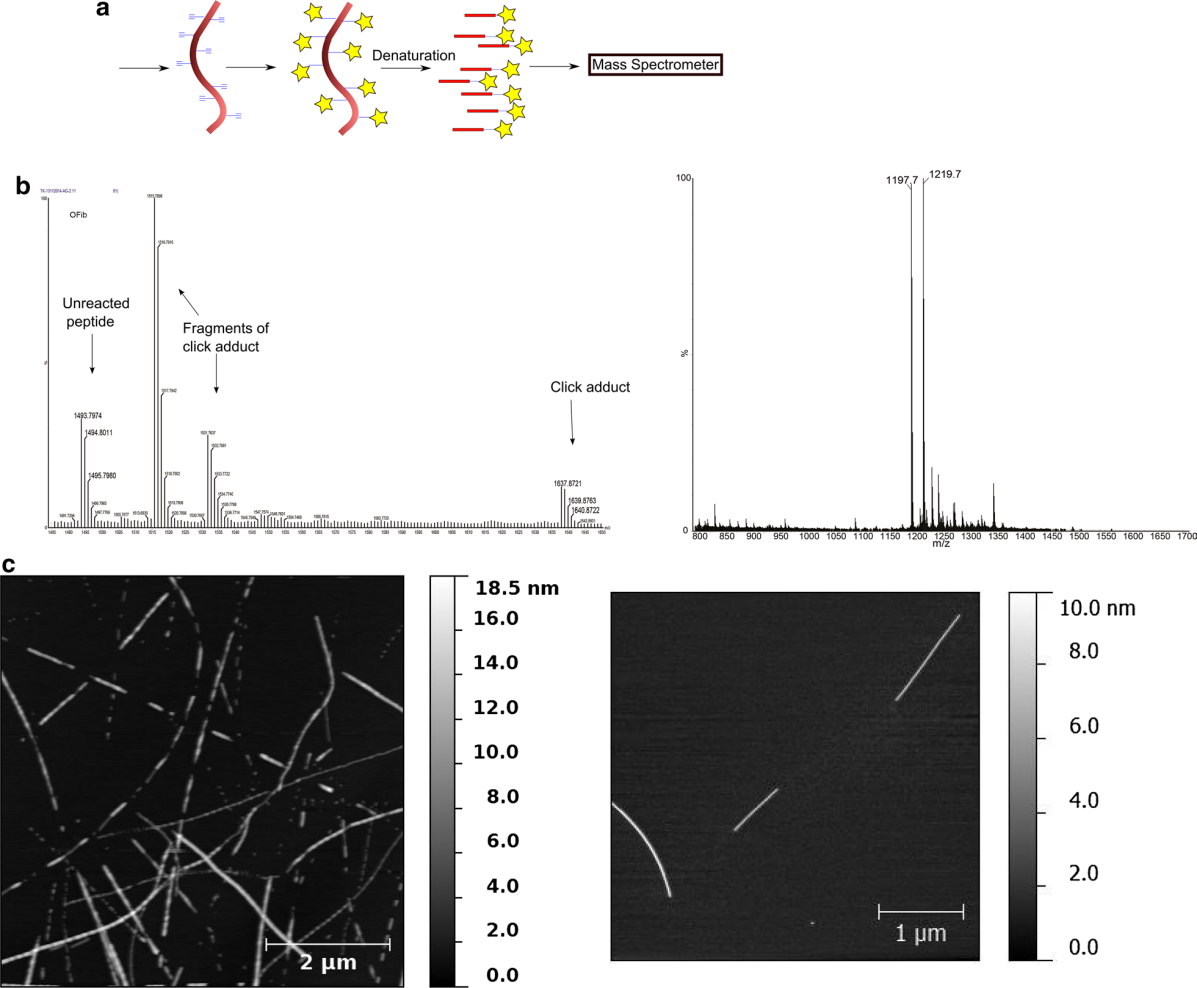
**a** Schema of the mass-spectrometry analysis strategy. **b** Mass spectrum of the TTR-A1 fibrils reacted with azidohomoalanine demonstrating the formation of the “click” adduct (left) and of TTR105-115 fibrils which did not react under the same conditions (right). **c** Atomic force micrograph of representative TTR-A1 fibrils (left) and of representative TTR105-115 fibrils (right)

We first carried out self-assembly of the TTR105-115 peptides into nanoscale fibrils and examined their morphology using atomic force microscopy. The micrographs show that their morphology and dimensions corresponded to that seen in the literature for TTR105-115 amyloid fibrils, being fairly straight fibrils with a length of several µm and a height of 6–8 nm. Figure 2 inset [12].

### Azidohomoalanine click experiments

In order to demonstrate the click reactivity of the self-assembled nanostructures, we exposed the fibrils to azidohomoalanine and isolated the nanostructures from any soluble material by means of ultracentrifugation. The fibrils were then denatured and the released peptides subjected to mass spectrometry analysis. The mass spectrum of the fibrils (Fig. 2) reacted with azidohomoalanine gave a peak at an *m/z* value of 1637.87, which corresponds to the calculated mass of the “clicked” adduct with azidohomoalanine. The major peak, at *m/z* = 1515.79, corresponds to the loss of the N-terminal tyrosine from this adduct, and the peak at 1531.76 corresponds to the loss of its sidechain. The peak at 1493.80 corresponds to unreacted peptide, either due to the reaction not going to completion or because the alkyne moieties in some of the peptide molecules were buried in the fibril and unable to react.

By contrast, fibrils made from the unfunctionalised ‘wild-type’ amide-terminated TTR105-115 peptide (sequence YTIAALLSPYS-NH_2_) did not react and the major peaks in their mass spectrum (Fig. 2) are at *m/z* = 1197.68, corresponding to unreacted peptide, and at *m/z* = 1219.7, corresponding to a sodium adduct. Note that the difference in *m/z* value for this peak compared to that for unreacted TTR-A1 is due to the unfunctionalised TTR105-115 peptide lacking the unreacted amino acid and the triglycine linker.

### Fluorescein click experiments

**Fig. 3:**
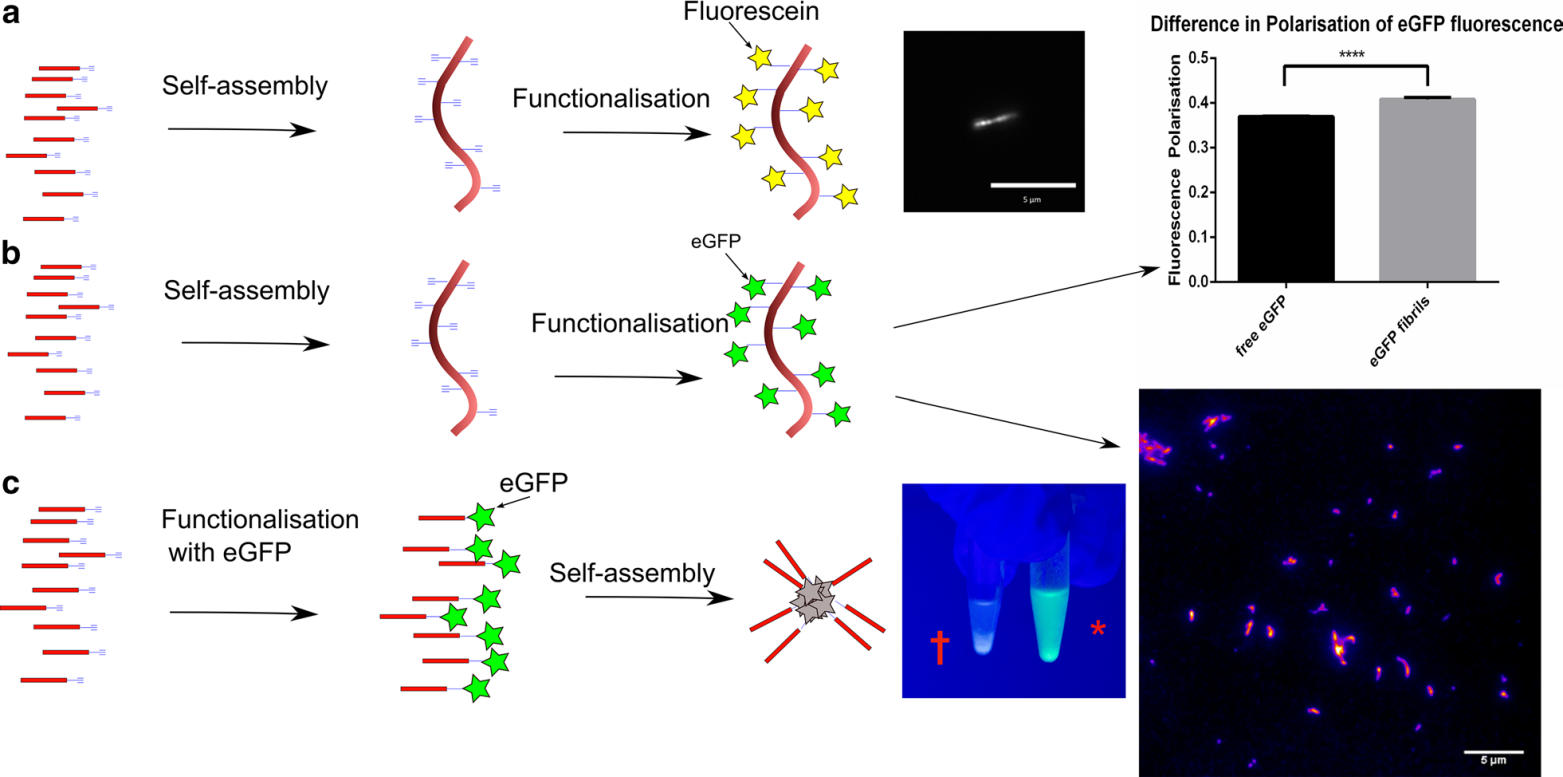
Reaction of fibrils and peptides with eGFP and fluorescein. **a** Fibrils assemble and can then be functionalized with fluorescein to give fluorescent fibrils, as shown in TIRF image. Scale bar 5 µm. **b** Fibrils assemble and can then be functionalized with eGFP to give fluorescent fibrils (†) as shown in TIRF image. Scale bar 5 µm. **c** Peptides functionalized with eGFP form aggregates that are not fluorescent (∗), as can be seen under UV illumination

We next repeated the functionalization process with a fluorescein analogue containing an azide group. The visible yellow color of the pellet after ultracentrifugation of the fibrils suggested that they had reacted to form a fluorescein adduct. Total internal reflection fluorescence (TIRF) microscopy (Fig. 3) showed fluorescent fibrils, confirming that fluorescein was localized to the fibril surface. This is due to a reaction between the alkyne functional groups on the peptide surface and the azide functionality on the fluorescein, resulting in fluorescein-functionalized and therefore visibly fluorescent amyloid fibrils.

### eGFP click experiments

In order to demonstrate the generality of our approach, we finally focused on coupling entire proteins to the nano-scaffold. To this effect, we used enhanced Green Fluorescent Protein (eGFP) as the cargo species. There was visibly more eGFP in the supernatant of the control sample than in that of the clicked fibrils, suggesting that eGFP had successfully been conjugated to the fibrils. Moreover, a visible precipitate had formed in the reaction mixture where aggregation took place after conjugation of eGFP to peptide. This reaction mixture was no longer fluorescent when exposed to UV light, whereas the clicked fibrils were (Fig. 3). Therefore, eGFP does not retain its fluorescence when exposed to the conditions of aggregation, possibly because it has itself aggregated.

TIRF micrographs show fluorescent fibrils (Fig. 3), whereas no fluorescent fibrils were visible in a “control” reaction carried out in the absence of copper sulfate and sodium ascorbate. The fluorescence polarization of eGFP that had reacted with fibrils was greater than that of free eGFP (Additional file 1: Figure S1). Although this difference is significant, it is smaller than might perhaps be expected given the very large size of the fibrils. This observation can be rationalized by considering that there is a long flexible “tether” of approximately 23 amino acids (the—GGGX section of the peptide followed by the tag on the eGFP from the pOPIN_F vector) between the fibril and the eGFP, and the N-terminal region of eGFP is also flexible [33]. Therefore, even bound eGFP has a high enough degree of freedom of movement to exhibit some fluorescence depolarization.

## Conclusions

In this paper, we have discussed and demonstrated a strategy to form nanoscale scaffolds that can be functionalized after their self-assembly has taken place. We showed that an approach that proceeds via click-reactive scaffolds allows a wide variety of molecular species to be used, including ones that are not compatible with the conditions required for self-assembly mediated nanoscale organization. We implemented this strategy using amyloid fibrils which were successfully formed from a peptide based on the TTR105-115 amyloidogenic sequence with the addition of a C-terminal alkyne functionality. The formed fibrils were shown to be capable of undergoing a “click” reaction with various azide-containing molecules, including proteins, which do not survive the conditions of fibril formation. These results open up the possibility of using amyloid scaffolds to organize a wide variety of cargo components, from small molecules to amino acids and proteins, for potential applications both in protein aggregation research and soft nanotechnology.

## Experimental methods

Unless otherwise stated, all reagents were used as received without further purification. Fmoc-protected amino acids were obtained from Novabiochem (Darmstadt, Germany) or AGTC Bioproducts (Hessle, UK) except for Fmoc-*O*-propargyl serine, which was synthesized as described in the Additional file 1. Tris(3-hydroxypropyltriazolylmethyl)amine (THPTA) was synthesized by Yu Heng Lau according to an established procedure [34].

### Peptide synthesis and fibril assembly

The peptide TTR-A1, with sequence YTIAALLSPYSGGGX-NH_2_ where X = *O*-propargyl serine, was synthesized on a CEM Liberty microwave peptide synthesizer (CEM Ltd., Buckingham, UK) on Rink amide resin (Novabiochem) according to standard Fmoc synthesis protocols [35] using HBTU as the activator and DIEA as the base. The peptide was manually cleaved from the resin using a 95:2.5:2.5 TFA:triisopropylsilane:water cleavage cocktail, precipitated with ether, and purified by HPLC on an Agilent 1260 Infinity system (Agilent, Stockport, UK) using a linear acetonitrile:water gradient before being lyophilized.

To induce fibril assembly, the lyophilized peptide was dissolved in a 10% solution of acetonitrile in water at a concentration of 10 mg/mL and incubated in a sonication bath to aid dissolution. This solution was then shaken at 600 rpm at 37 °C for 24 h to give a suspension of fibrils which were imaged by atomic force microscopy (AFM).

### Atomic force microscopy

Fibrils were diluted in milliQ water to yield a suspension with a final peptide concentration of 0.01–0.05 mg/mL. A 30 µL aliquot of the resulting diluted suspension was deposited onto a freshly cleaved mica surface and allowed to dry in air before being imaged using a JPK Nanowizard III AFM (JPK Instruments AG, Berlin, Germany) in intermittent contact mode with an uncoated HQ:NSC36 tip (MikroMasch, Wetzlar, Germany). Images were flattened, levelled and height medians of lines matched using standard techniques and the Gwyddion software [36].

### Click reaction with azidohomoalanine

The Huisgen reaction is a 1,3-dipolar cycloaddition reaction between an azide and a terminal alkyne to give a 1,2,3-triazole. The reaction is extremely regioselective, giving the 1,4-substituted triazole exclusively, and will tolerate a wide range of functional groups. The catalytic species is Cu(I), which is usually produced in situ by the reduction of a Cu(II) salt [37]. Fibrils were reacted with azidohomoalanine (Iris Biotech GmbH, Marktredwitz, Germany) using an adapted version of the method of Presolski et al. [38]. Azidohomoalanine hydrochloride (1.4 mM), TTR-A1 fibrils (1 mg/mL, 700 µM monomer equivalent), copper(II) sulfate (0.2 mM), THPTA (1 mM) and sodium ascorbate (10 mM) were dissolved in water (0.5 mL). All reagents were added as concentrated stock solutions in water—the copper(II) sulfate and THPTA were mixed before being added, and the sodium ascorbate added last. The reaction mixture was placed in a closed tube and shaken at room temperature for 24 h.

A 200 µL aliquot of the reaction mixture was ultra centrifuged at 313,000×*g* for 30 min using a TLA-100 rotor (Beckman Coulter, High Wycombe, UK) to isolate the fibrils. The supernatant, containing any unaggregated monomer, was removed by pipetting and the fibrils resuspended in 200 µL water. The resuspended fibrils were analyzed by liquid chromatography mass spectrometry (LC–MS) on a Waters Xevo G2-S instrument (Waters, Manchester, UK) using a C18 column. MS experiments were performed at a capillary voltage of 1900 V, cone voltage of 80 V and source offset voltage of 80 V. Spectra were processed using MassLynx V4.1 (Waters).

### Click reaction with fluorescein

Fibrils were reacted with 5-fluorescein amidite azide (FAM-5 azide, Lumiprobe, Hannover, Germany) (50 µM) using the same method as above. Again, a 200 µL aliquot of the reaction mixture was ultra centrifuged to give a visibly yellow pellet of fibrils. These were resuspended in 200 µL water before being diluted for imaging by total internal reflection fluorescence (TIRF) microscopy.

### Preparation of azide-functionalized GFP

A construct of eGFP in the pOPIN_J vector was a gift from Owen Burbidge (University of Cambridge). The protein was expressed in *E. coli* cells and purified using nickel-nitrilotriacetic acid (Ni–NTA) beads (QIAGEN, Manchester, UK) followed by size-exclusion chromatography on a HiLoad 26/60 Superdex 75 prep-grade column (GE Healthcare, Little Chalfont, UK). Purified protein was flash-frozen and stored at −80 °C.

The *N*-terminal amine of the eGFP was converted to an azide using the protocol of Schoffelen et al. [39]. This method reliably converts *N*-terminal amines to azides without modifying lysine residues. The protein was exchanged into 50 mM Clark and Lubs buffer at pH 8.9 using a NAP-5 column (GE Healthcare) to give a 71.8 µM solution. 240 µL of this was added to 227 µL of 50 mM Clark & Lubs buffer at pH 8.9 and 33 µL of a 2 mg/mL solution of imidazolyl-1-sulfonyl azide (synthesized by Yu Heng Lau, University of Cambridge, according to a literature procedure [40]). The mixture was shaken at 37 °C overnight, and the extent of diazotization checked by mass spectrometry.

### Functionalization of fibrils with eGFP

Fibrils were reacted with eGFP using a similar method to that above by adding fibrils and catalyst to the solution of azide-functionalized eGFP prepared in the previous paragraph. A control reaction was also carried out in the absence of the click catalyst mix. Again, after the reaction the fibrils were pelleted by ultracentrifugation and resuspended in water. As an additional control, the reaction was also carried out with unaggregated lyophilized peptide (10 mg/mL) instead of fibrils, with the addition of 10% acetonitrile at 37 °C. As the click reaction is faster than amyloid fibril formation, taking place over a timescale of tens of minutes [34] rather than hours [41], the intention of this control was to attempt to form fibrils after the peptide had been conjugated to eGFP.

The eGFP-functionalized fibrils were imaged by TIRF microscopy using a similar method to that used for fluorescein (see below). In addition, the fluorescence polarization of free eGFP and of eGFP clicked onto fibrils was measured using an LS-55 luminescence spectrometer (Perkin-Elmer, Beaconsfield, UK). Either a 7.18 µM solution of eGFP in Clark and Lubs buffer or a tenfold dilution of the pelleted and resuspended fibrils from the click reaction mixture were measured at 25 °C in a QS-284 quartz glass cuvette with an excitation path length of 10 mm and an emission path length of 2 mm, using an excitation wavelength of 488 nm and an emission wavelength of 509 nm. Quoted figures are an average of 10 measurements.

### TIRF microscopy

Fibrils were diluted tenfold and a 5µL aliquot was deposited onto chambered coverglass (80,827, µ-Slide, ibidi). Fibrils were allowed to settle for 10 min before adding 200µL PBS. Prior to imaging, the coverglass was cleaned using 1 M potassium hydroxide for 30 min before washing 3 times with ultrapure water (MilliQ). Fibril samples were imaged using a 100x/1.49NA objective (UAPON100XOTIRF, Olympus) on an Olympus IX71 inverted microscope with custom built TIRF illumination. The incident angle for TIRF was controlled by laterally translating the laser focus in the back focal plane of the microscope objective. EGFP was excited using a 488 nm diode laser (iBeam SMART, Toptica), with detection via a 525/30 nm bandpass filter (FF01-525/30, Semrock) onto an sCMOS camera (ORCA Flash 4.0, Hamamatsu). Image acquisition was controlled using Micro-Manager software [42] with 100–200 ms exposure times.

## Additional file

**Additional file 1.** Synthesis of *o-propargyl* serine, image processing details and fluorescence polarization results

## Abbreviations

AFM: atomic force microscopy
DIEA: *N*,*N*-diisopropylethylamine
*E.coli*: *Escherichia coli*
eGFP: enhanced Green Fluorescent Protein
HBTU: 2-(1H-benzotriazol-1-yl)-1,1,3,3-tetramethyluronium hexafluorophosphate
LC–MS: liquid chromatography mass spectrometry
PBS: phosphate buffered saline
TIRF: total internal reflection fluorescence
THPTA: Tris(3-hydroxypropyltriazolylmethyl)amine (THPTA)
TTR: transthyretin
