# Supplementary material for: Nanoscale click-reactive scaffolds from peptide self-assembly
Source: J Nanobiotechnology. 2017 Oct 6;15:70. doi: 10.1186/s12951-017-0300-7 (PMC6389178; doi:10.1186/s12951-017-0300-7)
Supplement: Supplementary file 1 — Additional file 1. Synthesis of o-propargyl serine, image processing details and fluorescence polarization results [file 12951_2017_300_MOESM1_ESM.docx]

**Supporting Information**

**Image processing**

TIRF microscopy images were processed using ImageJ (Wayne Rasband, National Institute of Mental Health, Bethesda, MD, USA). Fluorescence polarisation data (Figure 3b) were analysed using GraphPad Prism (GraphPad Software, La Jolla, CA, USA). Photograph in Figure 3c was processed using Adobe Lightroom (Adobe Systems, San Jose, CA, USA).

**Synthesis of Fmoc-*O*-propargyl serine**

*Reaction 1: Boc-O-Propargyl serine*

Boc-L-Serine (2.0g, 9.7mmol) was dissolved in DMF (100mL) and cooled on ice. Sodium hydride (60% dispersion in mineral oil, 935mg, 23.4 mmol) was added. After evolution of hydrogen gas stopped (about 20 minutes), propargyl bromide (80 wt% in toluene, 1.52 mL, 13.6 mmol) was added dropwise and the mixture stirred on ice for 15 minutes.

The mixture was allowed to warm to room temperature and stirred for 2 hours, then water added to destroy excess sodium hydride. The mixture was evaporated to dryness in vacuo and the residue dissolved in water. The aqueous layer was washed with ether and acidified with 1N potassium hydrogen sulfate.

The acidic aqueous phase was extracted with ethyl acetate (3x 80 mL), washed with water (80 mL) and brine (80 mL) and dried over Na_2_SO­_4_. The solvent was removed under reduced pressure, and the product purified by flash column chromatography with DCM:8%methanol:1%acetic acid as the eluting solvent. Boc-*O*-propargyl serine (1.84g including some solvent) was obtained as an orange oil.

*Reaction 2: O-propargyl serine*

The product of reaction **1** (1.84g) was dissolved in the minimum volume of diethyl ether. HCl (2.0M solution in diethyl ether, 10 mL) was added and the mixture stirred at room temperature for 2 hours. The progress of the reaction was followed by TLC.

On consumption of the starting material, the reaction mixture was filtered. *O*-propargyl serine hydrochloride (350 mg) was collected as an off-white precipitate, washed with ether, and dried under vacuum (desiccator).

The solvent was removed from the filtrate under reduced pressure. NMR showed that the filtrate still contained both *O*-propargyl serine and a significant amount of unreacted Boc-O-propargyl serine. Therefore, to ensure complete deprotection, the filtrate was dissolved in 10 mL TFA (20% v/v in DCM) and stirred overnight at room temperature.

The TFA salt of *O*-propargyl serine was precipitated with ether, giving 0.36g of a brown solid (1.40 mmol).

*Reaction 3: Fmoc-O-propargyl serine*

*O*-propargyl serine hydrochloride (**2**, 350 mg, 1.95 mmol) was dissolved in water (3 mL) and triethylamine (550 µL, 4 mmol). Separately, Fmoc-OSu (691.7 mg, 2.05 mmol) was dissolved in acetonitrile (2.2 mL) with gentle heating. The solutions were mixed and the pH raised to 9.0 with triethylamine. The solution was stirred for 30 minutes at room temperature, then filtered and the solvent removed under reduced pressure.

20% citric acid was added to the resulting sticky oil. The precipitate formed was filtered, dissolved in ethyl acetate, washed with 20% citric acid, water and brine, and dried (MgSO_4_). The solvent was removed under reduced pressure to give Fmoc-*O*-propargyl serine (446 mg, 1.22 mmol, 62.6%) as an off-white solid. As this was pure by TLC, further purification was not necessary.





Figure S1: Fluorescence polarisation of free GFP compared to GFP conjugated to fibrils.
